# Protein and Polysaccharide-Based Optical Materials for Biomedical Applications

**DOI:** 10.3390/ijms25031861

**Published:** 2024-02-03

**Authors:** Gianna Riviello, Brendan Connor, Jake McBrearty, Gianna Rodriguez, Xiao Hu

**Affiliations:** 1 Department of Physics and Astronomy, Rowan University, Glassboro, NJ 08028, USA; 2 Department of Biomedical Engineering, Rowan University, Glassboro, NJ 08028, USA; 3 Department of Biological and Biomedical Sciences, Rowan University, Glassboro, NJ 08028, USA

**Keywords:** protein, polysaccharide, optical material, optical fiber, waveguide

## Abstract

Recent advances in biomedical research, particularly in optical applications, have sparked a transformative movement towards replacing synthetic polymers with more biocompatible and sustainable alternatives. Most often made from plastics or glass, these materials ignite immune responses from the body, and their production is based on environmentally harsh oil-based processes. Biopolymers, including both polysaccharides and proteins, have emerged as a potential candidate for optical biomaterials due to their inherent biocompatibility, biodegradability, and sustainability, derived from their existence in nature and being recognized by the immune system. Current extraction and fabrication methods for these biomaterials, including thermal drawing, extrusion and printing, mold casting, dry-jet wet spinning, hydrogel formations, and nanoparticles, aim to create optical materials in cost-effective and environmentally friendly manners for a wide range of applications. Present and future applications include optical waveguides and sensors, imaging and diagnostics, optical fibers, and waveguides, as well as ocular implants using biopolymers, which will revolutionize these fields, specifically their uses in the healthcare industry.

## 1. Introduction

**Figure 1:**
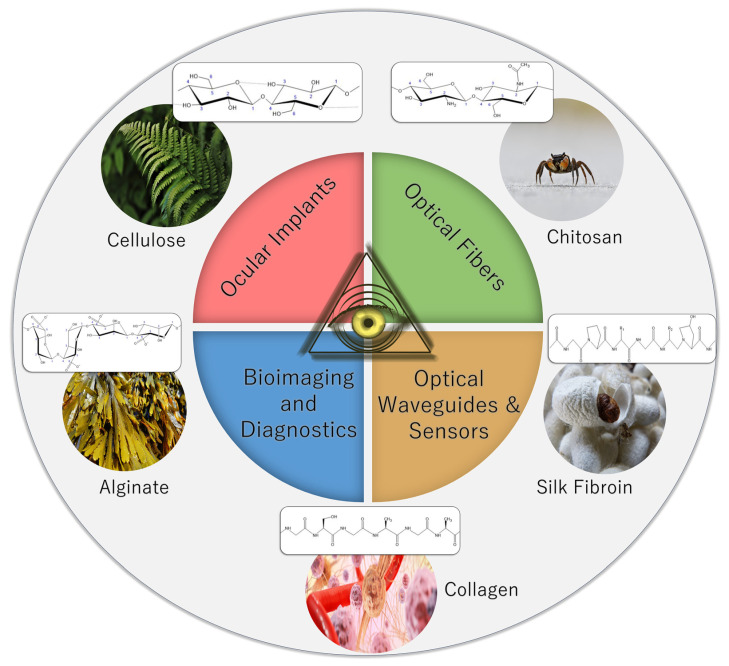
Sources and structures of proteins and polysaccharides and their common optical applications (photos obtained from Pixabay.com under the Pixabay Content License).

Biopolymers have always occupied an important role throughout human history. Our collective reliance on these materials for necessities such as clothing was as prolific among our ancestors as it currently is for modern humans. Today, biopolymers have found extensive applications in various medical contexts, including drug delivery and tissue engineering [1,2,3,4,5]. These materials are rapidly emerging as a probable successor to synthetic polymers because these are macromolecules made from natural sources, with the most abundantly researched being proteins and polysaccharides [1]. Their advantage and expanding momentum are their intrinsic derivation from biological systems versus mimicking them synthetically, overcoming the defects from imperfect replication of highly complex biological systems, specifically in the field of biomaterials science. Particularly, they are emerging with potential in the domain of optics and optical elements, which are elements used for light transmission, detection, and emission that serve many purposes in the medical field, specifically with treatments, therapies, and sensors [1]. Currently, plastics, such as poly (methyl methacrylate) or polystyrene, or glass, such as silica glass, are commonly used in these elements due to their high optical transmittance, and they have been employed in this industry since its inception [2,3]. Their poor compatibility with the body upon degradation and mismatch of mechanical properties of living tissues leaves them unable to serve as long-term implants, and therefore biopolymers are emerging not only as a replacement but an enhancement of optical technology [4,5]. In addition, biopolymers are obtained from natural resources, making them a sustainable replacement for synthetic polymers, especially due to the manufacturing processes of some synthetic polymers being harsh on the environment, mainly due to oil-based production [1,2,5,6]. Therefore, the naturally obtained and derived biopolymers are progressively examined as more sustainable, efficient, and potent alternatives to synthetic materials for optics and optical devices, presenting a revolutionary advancement in biomaterials science and engineering. However, the success of optical biopolymers hinges on far more than their theoretical benefits or demonstrated efficacy in medical devices. To truly succeed in a global market, biopolymers may need to rival or usurp synthetic polymers. Given the inexpensiveness of the raw materials needed to produce biopolymers, it seems likely that biopolymers may be not only clinically viable, but commercially viable as well. An overview of protein and polysaccharide biopolymers and their typical optical applications is shown in Figure 1.

Biopolymers are currently being researched in optics for medical devices in a variety of applications, including waveguides for guiding light, biosensors, optical fibers as probes for continuous patient monitoring, light therapy inside patients, or wearable devices [2,3,6]. These applications are focused on proteins and polysaccharides, including cellulose, chitosan, alginate, collagen, silk fibroin, and gelatin [1,7,8,9,10,11,12]. Of all the biopolymers listed, silk fibroin and nanocellulose are most frequently cited in the literature due to their high uniformity and optical clarity [3,6,13]. These materials can also form the basis for more complex structures of optical biomaterials. Biopolymers exhibit potential in the field of optics, specifically optical devices and elements in biomedical science, because they possess the favorable combination of optical properties, biocompatibility, biodegradability, and sustainability, a combination that some synthetic polymers lack. Their optical properties such as optical transmittance, light guiding efficiency, and refractive index replicate or surpass those of most synthetic polymers [1,2]. In addition, they also possess low absorbance and scattering properties that increase their efficiency in light transmission [2]. Concurrently, these properties make them ideal for optical devices that can efficiently and effectively transfer light, even inside the human body, a realm where most modern research is focused. The main advantages of protein and polysaccharide biopolymers over some synthetic polymers as biomaterials are their combined properties of biodegradability and biocompatibility [1,5,7]. Each of the materials discussed here is known to be far less likely to cause an adverse reaction upon implementation in the body, whereas some synthetic polymers can elicit an immune response in the body upon degradation. Biodegradability adds yet another positive attribute to biopolymers due to the elimination of surgical removal post-implantation, and research in biopolymer science represents a significant trend toward this end with implantable biopolymers [14,15]. In addition, plastics often degrade into highly toxic microplastics, eliminating their potential as both biodegradable and biocompatible biomaterials [5]. Together, these properties with their low relative cost present protein and polysaccharide biopolymers as cost-effective and safe medical devices [14].

In addition to their optical and material properties as discussed, biopolymers present favorability over synthetic polymers due to their customizable fabrication methods, giving them the ability to be combined with other materials to essentially customize their physical, mechanical, and chemical properties [16]. They can be tailored to specific needs or design constraints, most often matching properties for a specific tissue in the body or a specific application. For example, functional groups can be added to them to customize their refractive index, interface with the outside environment, or their assembly [1,7,10]. In addition, if implanted, their mechanical properties can be designed to match those of the surrounding tissue to prevent tissue damage [3]. This customizability allows for the optimal integration into the body and functionality. Nanoparticles are commonly used in research to achieve this end, with the biopolymers used as a matrix for them [11,12,13,14]. For example, carbon, metals, and minerals can be used to control thermal and electrical conductivity in protein and polysaccharide biopolymers [7]. Current fabrication processes vary, but they commonly include making films, hydrogels, 3D printed structures, molds and templates, and certain matrix formations and crosslinking for nanoparticle deposits [1,4]. Research aims to optimize these processes in multiple ways to overcome some of the faults that exist with using biopolymers, such as their instability. As they degrade, their properties can change [4,14]. In addition, methods to obtain and purify biopolymers can damage their performance in vitro and in vivo [13]. Therefore, there are still processes and challenges with implementing proteins and polysaccharides for optical purposes that research aims to explore and address.

Current research is focused on looking for methods to optimize the utilization of biopolymers for many applications, including optical elements for devices and implants. This review outlines commonly used protein and polysaccharide biopolymers and their properties, important optical properties and relevant theory, fabrication of these biopolymers, and their applications in optics.

## 2. Optical Biopolymer Materials

### 2.1. Polysaccharide Biopolymers

**Figure 2:**
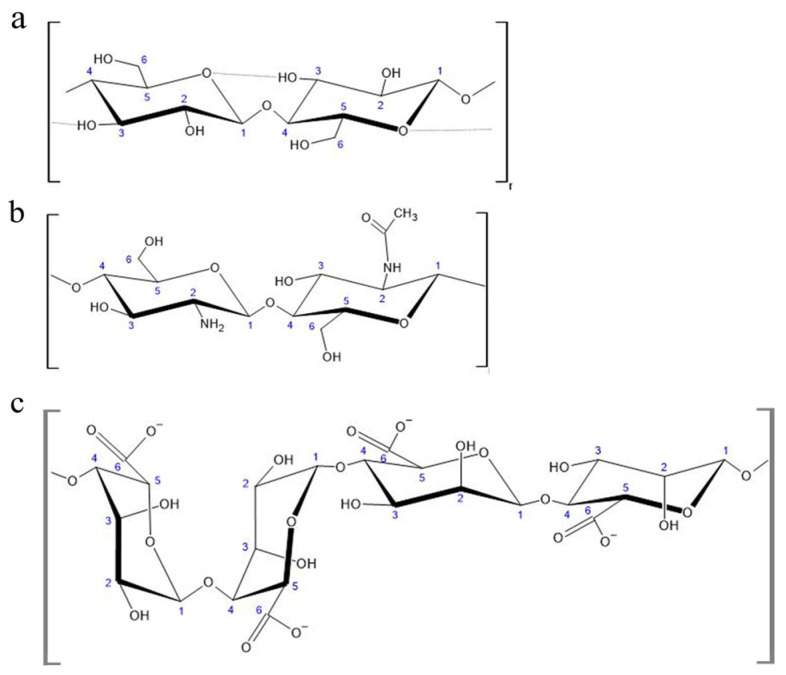
Chemical structures of polysaccharide biopolymers: (**a**) cellulose, (**b**) chitosan, (**c**) alginate. Numbers are used to indicate the position of carbon atoms within the individual sugar units (monosaccharides).

Polysaccharides are biopolymers consisting of monosaccharide or disaccharide units linked together via dehydrated glycosidic bonds, and they can be classified by the presence of different types of monomers in their structures; homopolysaccharides only consist of one single type of monomer while heteropolysaccharides can have multiple different monomers [7]. In this review, three polysaccharides will be discussed: cellulose, chitosan, and alginate. The chemical structures of these polysaccharides can be seen below in Figure 2.

#### 2.1.1. Cellulose

One of the most plentiful biomaterials on the planet, cellulose is a biopolymer consisting of long chains of β 1→4 linked glucose monomers with the chemical formula of (C_6_H_10_O_5_)_n_ that naturally bundle together through several intermolecular forces, such as hydrogen bonds, van der Waals interactions, and dipole moments, into fibrils, the basic unit of cellulose polymers. Upon fibril formation, they connect to form microfibrils, which in turn are organized into a tertiary network-like structure called nano-fibrillate cellulose (NFC). This high degree of intermolecular connectivity renders cellulose fibrils rigid, allowing the material to conduct its primary purpose as reinforcement and protection for plants, bacteria, algae, and a type of marine filter-feeding invertebrate called tunicates.

Due to variability in its assembly, cellulose can have a crystalline or amorphous structure, in addition to a combination of the two structures, depending specifically on preparation methods and source of the cellulose used. It can also be refined into nano paper, sheets of NFC that are thinner in both diameter and spacing than visible wavelengths of light, leading to high degrees of optical transparency [7,8,9]. Purely crystalline cellulose can be extracted to form cellulose nanocrystals (CNCs), of which there are four polymorphs, named I through IV, respectively. Cellulose I is found naturally produced by living organisms, is thermodynamically metastable, and can be further broken down into two structural types: I⍺ (triclinic) and Iβ (monoclinic). These two types are miscible at various ratios depending on the source of the cellulose, with varying amounts of each affecting the structure’s bulk properties. Higher levels of Cellulose I⍺ can be found with algae and bacterial sources while higher levels of Cellulose Iβ are found in plant and tunicate cell walls, though Cellulose I⍺ can be converted to Iβ using heat and solvent treatments. Cellulose II has a monoclinic crystalline structure and is produced by either dissolving and recrystallizing Cellulose I or by treating it with sodium hydroxide. Cellulose III can be formed by treating either Cellulose I or II with ammonia. Lastly, Cellulose IV is created by further treating Cellulose III with heat [9].

#### 2.1.2. Chitin and Chitosan

Chitosan is a heteropolysaccharide consisting of glucosamine and N-acetyl glucosamine linked by β 1→4 glycosidic bonds derived from the chitin present in the exoskeletons of crustaceans, the cuticles of arthropods, and the cell walls of fungi. This process involves either deacetylation under alkaline conditions or the use of a chitin deacetylase to deacetylase half or more of the glucosamines in the chitin, resulting in its solubility in aqueous acidic solutions as the amine group on the second carbon of the glucosamine will protonate. Its variable solubility depending on pH allows the use of chitosan in aqueous, gel, and solid contexts, as well as serving the purpose of a flocculant when a solution is returned to a neutral or basic pH. The solubility of a given sample of chitosan depends heavily on the distribution of acetyl groups and the molecular weight of the sample. In solid form, chitosan is semicrystalline and has many different polymorphs. Acetyl groups are hydrophobic but can be protonated by a sufficiently acidic solution to allow the polymer to dissolve [10,11]. In addition to its solubility and structural properties, chitosan is known to form complexes with various metals due to the acetyl groups present on the chain [11]. Azofiefa et al. reported that chitin absorbs negligible amounts of light and that the refractive index of chitin is dependent on the structure of the specific sample. Despite this, many researchers tend to assume a general refractive index for chitin of 1.56 with optical constants between 250 and 750 nm. The aforementioned value for the refractive index of chitin was provided using assumptions regarding the structure of chitin layers informed by electron microscopy [17].

#### 2.1.3. Alginate

Alginate is an unbranched polymer derived from brown seaweed or bacteria that consists of 1→4 linkages of β-D-mannuronic acid and α-L-guluronic acid. These two monomers can be found arranged in an alternating pattern, called MG-blocks, or alone, called M-blocks and G-blocks, respectively [18,19]. Alginate produced by brown seaweed differs from that produced by bacteria in two critical ways: first, the length and distribution of the various blocks differs between the two sources, and second, bacterial alginate is typically acetylated while seaweed alginate is not. Alginate is most prominently used as a gelling agent, which gives it uses in fields such as food production, biomedical science, and optics. A study by Stokke et.al. indicated that the strength of an alginate gel is correlated with an increase in G-blocks [18].

### 2.2. Protein Biopolymers

**Figure 3:**
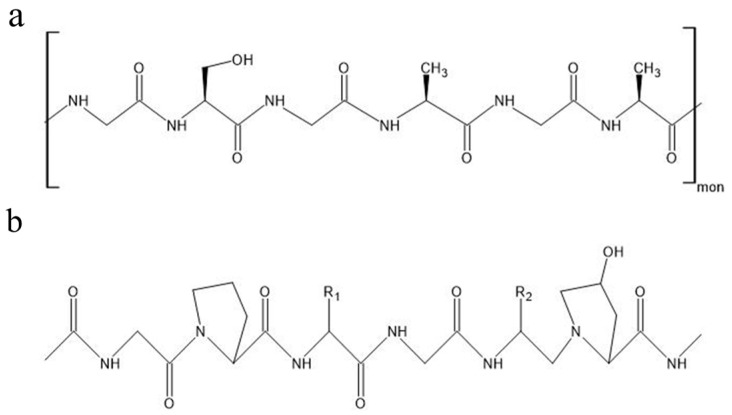
Chemical structures of protein biopolymers: (**a**) silk fibroin, (**b**) collagen type 1.

Proteins are among the most complex of biopolymers, consisting of a sequence of amino acids that fold and form structures with secondary bonds and intermolecular forces. These polypeptides are themselves organized into a tertiary protein structure that governs the overall shape, function, and properties of the protein. Many proteins also have a quaternary structure that describes the form of proteins that are assembled from multiple polypeptide chains. These four levels of protein structure determine the various properties of each protein [7]. In this review, three polypeptide biopolymers will be discussed: silk fibroin, collagen, and gelatin (Figure 3).

#### 2.2.1. Silk Fibroin

Silk fibroin is a polypeptide produced by silkworms (genus *Bombyx*) that possesses incredible mechanical strength despite its light weight, consisting of a combination of large, repetitive amino acid sequences that are arranged into crystalline β-pleated sheets and sequences of nonrepetitive amino acids organized into semiamorphous structures like helices and spirals. Commonly, the crystalline β-pleated sheets would consist of repeating sequences of alanine, glycine–alanine, and glycine–alanine–serine, while the amorphous regions could have a random makeup. This combination provides the material with both strength and flexibility, and modifications to the proportions of crystalline and semicrystalline structures can alter the properties of a given sample of silk fibroin [20,21].

Silk fibroin from *B. mori* is particularly used in optical applications due to the polymorphism of the material. Silk from *B. mori* can be processed and split into its crystalline and amorphous components by taking advantage of the difference in solubility between the two portions: amorphous regions are much more soluble than crystalline [21]. This process also allows researchers to insert various materials, both organic and inorganic, into the structure. In the field of optics, this is commonly seen with the addition of fluorescent quantum dots inside of the silk to form a nanocomposite that maintains the fluorescence without compromising the silk or any other potential additives like nanoparticles [7]. Additionally, silk fibroin has been demonstrated to have high thermal stability and thermoplasticity because heat causes the amorphous segments to crystallize and expel nonstructural water. Silk structure has also been shown to be highly adaptive to various environments, conditions, and situations due to its heavy structural reliance on both intramolecular and intermolecular hydrogen bonds [21]. Lastly, the use of silk fibroin has optical applications due to its high refractive contrast in most environments. *B. mori* silk fibroin demonstrates a refractive index of 1.54 at 633 nm. This can be improved further by embedding the silk with refractive material such as titanate nanosheets to give the silk an adjustable refractive index [7].

#### 2.2.2. Collagen and Gelatin

Collagen is a fibrous protein characterized by triple helix structures present in the connective tissue of nearly all living organisms. While 29 types of collagens have been described and studied, only Collagen types I, II, III, V, and XI are known to form collagen fibers comprised of three ⍺ chains composed of glycine in every third amino acid position, with other amino acids, like proline and 4-hydroxyproline, commonly taking up the other two positions. All 29 collagen types are formed from a unique combination of three of the 25 known unique ⍺ chain conformations [22]. In the field of optics, collagen is primarily utilized in the form of gelatin, a material formed by the partial hydrolysis of collagen by an acid or alkali. Gelatin is a water-soluble gel that is less mechanically impressive than collagen but has other properties that present its potential in optics. For example, gelatin is transparent and has a refractive index of 1.536 at 633 nm [7]. In particular, gelatin is commonly used as a medium to suspend silver halide solution for creating photographic and holographic film. This medium, called silver halide sensitized gelatin (SHSG), displays high exposure sensitivity, a broad spectral recording bandwidth, high diffraction efficiencies, beneficial signal-to-noise ratios, and high light stability [23].

## 3. Optical Theory in Biomaterials Science

### 3.1. Refractive Index

In optical elements, the first variable essential for optical fabrication methods and design is the refractive index, which quantifies the ability of a medium, such as a protein or polysaccharide biopolymer, to bend light. Equation (1) defines this quantity:

(1) $$n=\frac{c}{v}$$

where *c* is the speed of light in free space and *v* is the velocity of light in the medium. The refractive index of a medium is always greater than 1 since the velocity of light in a medium is lower than that in a vacuum [24]. This simplistic description of refractive index, while sufficient for basic understanding, can be further expanded upon through the knowledge that refractive indices exist as complex numbers. The full mathematical definition of the refractive index is given below in Equation (2):

(2) n=n+ik

where *n* is the phase velocity, and *k* is the absorption coefficient or extinction coefficient. The extinction coefficient is a measure of the extent to which light is absorbed at different wavelengths [25].

### 3.2. Snell’s Law of Refraction

Essential to all optical design and theory is Snell’s law of refraction, which is shown in Equation (3) below:

(3) n′ sin(i′)=n sin(i), 

where *n* and *n*′ are the angles of incidence before and after passing through media, respectively, *I* is the angle of incidence, and *i*′ is the angle of the light ray post-refraction, particularly useful for evaluating transitions between different biopolymer materials in an optical application [26]. In addition, Fermat’s principle describes and augments this theory further, stating that light often tends to travel in the swiftest way possible, regardless of media type. Combined together, these optical principles permit ray tracing and further optical property evaluation, and, with it, the development of optimal optical biomaterials [26]. It may be worth noting, however, that it is not uncommon for instances to arise where the path taken by light does not result in the fastest possible path. This is especially true for the reflection and refraction of light off of spherical and cylindrical surfaces, as demonstrated by Se-yuen Mak [27].

### 3.3. Optical Fiber and Waveguide Theory

Of the diverse applications of protein and polysaccharide biopolymers in the optical industry, one of the most abundant is the fabrication of optical fibers and waveguides. In their fabrication, an essential principle is internal reflection, which is the containment of light. The core possesses a higher refractive index than the cladding. Light that enters this core below a certain angle, called the acceptance angle, reflects internally, remaining in the core as it travels. Above this angle, light is not refracted enough to remain in the core and is lost to the cladding. Completely internal light travel, or total internal reflection, where light remains entirely in the fiber with no loss is the objective of optical waveguides and fibers [5,24]. In defining the angles where total internal reflection occurs, the numerical aperture of the optical fiber is used to this end. Equation (4) is used:

(4) $$NA=sin\;\left(i_{max}\right)={\left(n_{1}^{2}-n_{2}^{2}\right)}^{1/2}\;$$

where *NA* is the numerical aperture, *i_max_* is the acceptance angle, *n*_1_ is the refractive index of the core, and *n*_2_ is the refractive index of the cladding.

The difference in refractive indices between the core and cladding materials is also particularly useful in calculating the refractive index difference, which is given by Equation (5):

(5) $$\Delta =\frac{\left(n_{1}^{2}-n_{2}^{2}\right)}{2n_{1}^{2}}$$

where *n*_1_ and *n*_2_ are the refractive indices of the core and cladding materials, respectively [28]. As demonstrated by Equations (4) and (5), refractive index is an important parameter for fiber design. The difference in refractive index between the core and cladding dictates the numerical aperture of the fiber, meaning that a higher difference allows for higher acceptance angles in the fiber, thus increasing the ability of the fiber to refract light internally [5,28].

Each biopolymer possesses an optimal wavelength of operation and a point of minimum attenuation loss that dictates the optimal distance of material to be used and the fabrication methods based on the requirements of the application [5]. In calculating attenuation loss, there are three factors to measure, including molecular vibration harmonics loss, Rayleigh scattering, and absorption loss from electronic transmission. Equation (6) is used:

(6) $$\alpha =\alpha_{v}+\alpha_{R}+\alpha_{e}$$

where $\alpha_{v}$ is the harmonics of molecular vibration loss, $\alpha_{R}$ is Rayleigh scattering loss, and $\alpha_{e}$ is electronic transmission loss [5]. It is crucial to minimize this loss to maintain efficient light transmission in optical fibers and waveguides.

## 4. Fabrication Methods for Optical Devices

Biopolymers in optical biomaterial applications are extracted, further modified and fabricated from various natural forms and methods due to the crude nature of biopolymers. Different methods of fabrication result in different molecular structures and properties, varying in each material, and fabrication methodology can be split into two categories: “top-down” and “bottom-up”. For the basic fabrication of biomaterials, the “top-down” approach is utilized, where biomaterials can be formed by stripping down more complex entities and structures into their basic components for further use and customization [16]. This directly contrasts with a different method known as the “bottom-up” approach, where materials are formed one molecule at a time [16]. Each process has benefits and drawbacks, depending on the research objective or application of the optical biomaterial, but independent of the fabrication approach, key elements in the fabrication process for most biomaterials include chemical complementary properties and structural compatibility [16]. General methods of biopolymer fabrication include various forms of mold casting and wet spinning, evaluated upon polymer loss, optical wavelength, and additional optical, chemical, and mechanical properties. Table 1 shows a summary of the fabrication methods and their benefits.

### 4.1. Thermal Drawing

**Figure 4:**
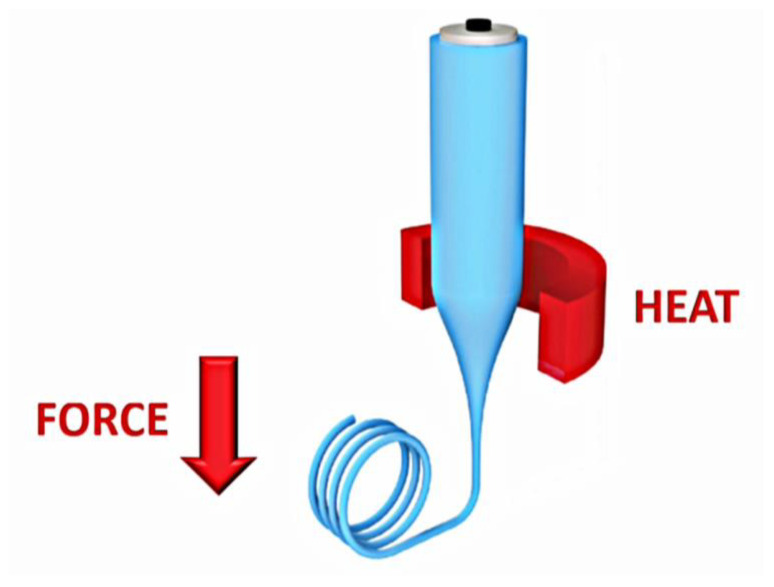
Schematic of the thermal drawing process. The preform of the optical fiber is heated to an appropriate temperature, and a downward force draws the fiber downward until the desired size is reached [29] (reproduced with permission from John Wiley and Sons, 2021).

One of the most frequently used and widespread manufacturing techniques for optical fibers is the heat drawing process, initially developed for silica glass optical fibers but increasingly used for biodegradable optical fiber development [29]. The thermal drawing technique utilizes a preformed optical fiber of a specified geometry, the first of two stages in the fabrication process. This preform can be prepared through several techniques, including thin-film rolling, 3D printing, or injection of molten polymers, depending on whether the desired optical fiber has multiple layers and has a hollow inside [29]. The second step of the thermal drawing process involves drawing down the preform into an optical fiber using a specific heat draw tower, shown in Figure 4. In this process, the fiber is drawn from the heating tower at a constant speed, reducing the diameter of the fiber to the chosen size [29].

Examples of the thermal drawing method were executed by Dupuis et al., discussing various optical fiber fabrication techniques followed by thermal drawing. In their study, they fabricated fibers composed of cellulose acetate, cellulose butyrate, hydroxypropyl cellulose, poly(ε-caprolactone), and poly(L-lactic acid) using several different techniques, fiber geometries, and cladding, with the aim of an efficient total internal reflection mechanism, a key characteristic of optical fibers [29]. Dupuis et al. then utilized the heat drawing process and reported attenuation losses ranging from 1 dB/cm to 10 dB/cm at a wavelength of 633 nm, comparable to the existing properties of existing plastic optical fibers [29].

### 4.2. Mold Casting

Casting is the process of pouring molten polymer into different casts and molds for the desired form or shape of the material. A process typically used for glass-based polymers, it is also applied towards biopolymers. Upon dissolving in a solvent, biopolymers are poured into a mold, typically cylindrical in shape [2]. The biomaterial is further treated to remove excess water or any other possible contaminants, and heated and melted into a liquid glass form to be drawn into an optical fiber [2,30]. Yet another different method for casting involves the solution casting of the initial polymer in a solvent, such as chloroform, followed by drying in a room-temperature environment or in a vacuum, allowing it to cure. This alternative method provides a relatively simple and straightforward method of fiber fabrication commonly suited for the production of large quantities of fibers at varying lengths [2].

**Figure 5:**
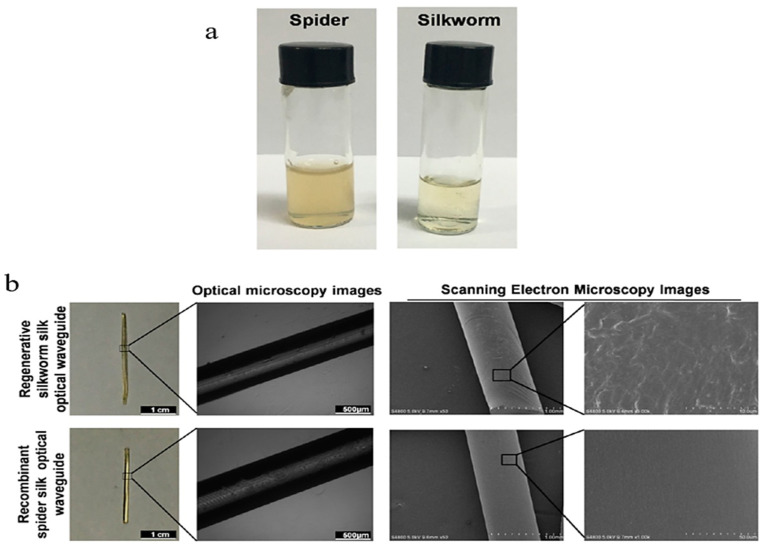
(**a**) Images of the casting process, shown by Qiao et al. for spider and silkworm silk. In this process, the silk is dissolved in a solvent, heated to eliminate water, and stored overnight. Finally, it can be removed from its mold. (**b**) Optical and SEM microscopy images of the fabricated optical waveguides; Scale bars = 1 cm (camera), 500 μm (optical microscopy), 1 mm (low magnification SEM) and 10 μm (high magnification SEM). [30] (reproduced with permission from American Chemical Society, 2017).

Qiao et al. performed a study analyzing the performance of genetically modified *N. clavipes* spider and *B. mori* silkworm optical waveguides, fabricated by mold casting. Each sample was dissolved in 1,1,1,3,3,3-hexafluoroisopropanol (HFIP) in a 400 mg/mL solution, shaken, and stored at room temperature overnight until proteins dissolved. Upon pipetting each silk solution in a polytetrafluoroethylene mold, the samples were heated at 60 °C for a week to eliminate water and HFIP from the fiber mold, from which they were removed, as shown in Figure 5a [30]. Images of the completed fibers are shown in Figure 5b. The refractive indices of the spider- and silkworm-derived waveguides were found to be n = 1.70 and n = 1.52, higher than that of biological tissue, demonstrating the feasibility of this fabrication method. This result, combined with its low optical loss, shows that this fabrication method can efficiently produce efficient optical waveguides or contact lenses [30].

### 4.3. Dry-Jet Wet Spinning

**Figure 6:**
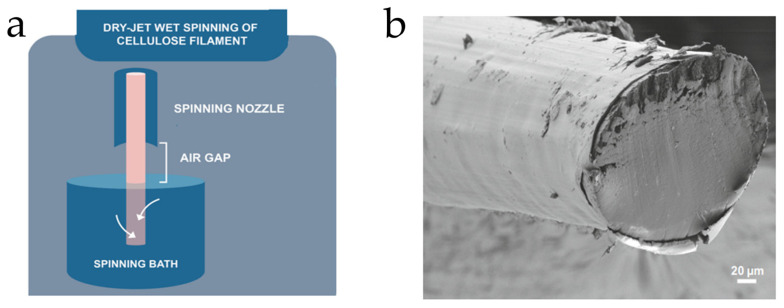
(**a**) A schematic of the dry-jet wet spinning process. The polymer-based solution is injected into the spinner jet. It makes its way to the coagulation bath, where nonsolvent removes the solvent from the polymer-based solution. The material is guided, stretched, and dried in the air gap to form an optical fiber or waveguide. (**b**) SEM image of an optical fiber with cellulose core and cellulose acetate cladding [32] (reproduced with permission from Springer Nature, 2020).

The process of dry-jet wet spinning involves the use of a syringe pump to inject a polymer-based solution into a spinner jet, which directs the solution into a coagulation bath filled with a nonsolvent, which removes the solvent from the polymer through diffusion or chemical reaction, shown in Figure 6a. Upon completion of the coagulation process, several wheels guide and stretch the material while simultaneously drying it to produce a desired shape with increased strength and stiffness. The process offers an advantage due to its relatively low operating temperatures, helping to preserve material properties, especially optical properties [31]. The drying of the polymer exhibits the uniqueness of the dry-wet spinning process from typical wet spinning because it results from a gap between the wheels and equipment for air exposure, which affects the development of the biopolymer fibers, as they swell when exposed to the air and narrow as wheels guide them. This process results in the development of optical fibers with various properties, depending on the solution used in the coagulation bath [31].

In a study conducted by Orelma et al., an optical fiber composed of cellulose was fabricated using dry-jet wet spinning, with a core made of dissolved cellulose dry-wet jet spun into the water and cladding made of cellulose acetate [32]. The cellulose was dissolved in the solvent, filled in a plastic syringe, and passed through the nozzle into the water coagulation bath, where the filaments remained for two hours and dried under tension. An SEM image of the fiber is shown in Figure 6b.

### 4.4. Formation of Hydrogels

Hydrogels are networks of polymers which are highly hydrophilic, viscoelastic, and self-supporting mesh which have a range of properties to be utilized on living tissue [34]. Hydrogels can swell with water and react independently based on environmental factors such as pH. Their ability to absorb high water content is provided by their hydrophilic groups such as -NH_2_, -COOH, -OH, CONH_2_, -CONH, and -SO_3_H [34,35]. The soft and malleable nature in addition to the high water content of hydrogels allows them to mimic human tissue well, making them ideal in technology for contact lenses and ocular advancements. The fabrication of hydrogels occurs through the molecular crosslinking of various monomers to form 3-dimensional complex biopolymer matrices. Methods of crosslinking hydrogels can be physical or chemical processes; such examples include hydrogen bonding, crystallization, and protein interactions. Hydrogen bonding is one of the easiest methods for forming hydrogels; as many molecules present in the biopolymers can form hydrogen-based interactions, this allows the hydrogels to self-assemble due to their molecular structures. In crystallization, the biopolymer chains synthesize hydrogels through the adjustment of their crystallization temperature, through freezing and thawing of the biopolymer solution [35]. Protein interaction fabrication occurs via the addition of certain proteins capable of antibody–antigen interactions with the biopolymers. The assistance of these proteins allows the hydrogels to form their 3D structures on a molecular level. By using distinct structuring and patterning, hydrogels can have specific optical interactions which can alter when the hydrogels interact with their environment.

**Figure 7:**
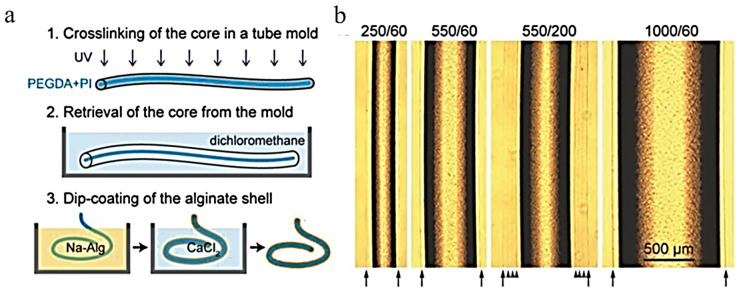
(**a**) A breakdown and schematic of the hydrogel formation process, from the crosslinking of the core in a tube mold to obtain the desired shape. Then, the retrieval of the core and the dip coating of the alginate shell. (**b**) Imaging of the hydrogels and the varying diameters they can be formed into; sizes include 250/60, 550/60, 550/200, and 1000/60; arrows show the clad-water boundary, and arrowheads mark the interfaces of layered alginate from multiple dip coatings. [33] (reproduced with permission from John Wiley and Sons, 2015).

Alginate is typically used in the formation of hydrogels. Choi et al. produced a biocompatible optical fiber formed from poly(ethylene glycol) (PEG) and alginate hydrogels that can be filled with functional nanoparticles and molecules [33]. Choi et al. formed a core–clad structure with a step-index profile with PEG as the core and the alginate as the cladding, demonstrated in Figure 7a. Biocompatible hydrogels were used in the formation of the core and cladding layers, which allowed for the addition of various fluids and nanoparticles to impact the fibers [33]. The cores were fabricated using a two-step process, starting by using a platinum-cured silicone mold to form the core. The hydrogel polymer was injected into the mold then cured using ultraviolet lighting to crosslink, then finally dipped into a Na–Alg solution to form the alginate shell coating, as shown in Figure 5a. These cores can vary in length and shape because of this process, shown in Figure 7b, as they come in sizes of 250/60 up to 1000/60. The process took about 2 h in total and was a reproducible and scalable procedure [33].

### 4.5. Extrusion and Printing

Three-dimensional printing is a generalized process for forming fibers and networks of structures using a molten substance. It can be customized to various applications depending on the materials used and the technology required. This can range from extrusion-based printing for most polymers, to ink-writing-based printing used in the fabrication of silk-printed optical waveguides [2,36].

In the process, a molten polymer is forced into a die with a specified pattern to form a desired shape, a process commonly used for spider silk formation into optical waveguides. For continuous extrusion, a molten mixture of monomers, initiators, and additives are extruded in a continuous manner during which polymerization occurs inside a reactor [2]. Typically, batched extrusion is performed, where multiple batches of polymers are processed at once, which permits a direct and simplified polymer fiber fabrication technique, as this allows for more polymer fiber processing completed in less time for overall higher productivity. After the full conversion of starting materials, the reactor’s temperature is raised to form a polymer melt. Extrusion-based molecules use high amounts of force and temperature to dispense material through a micronozzle, with their shape and form of the fibers depending on the deployed extrusion die. This setup also provides additional customizability in the fabrication process, as in a die with an appropriate pattern used for manufacturing air-holed structures in any polymer-based materials [2]. While different printers and machines may have variety in the exact process for printing, extrusion-based processes obey the same principles for transforming a nonsolid polymer to a 3D fiber or other shape, which can be altered depending on the chemical composition and structure of the material.

**Figure 8:**
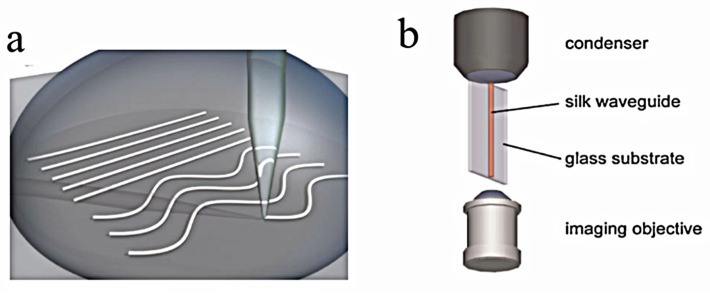
(**a**) The schematic and process of direct-write printing of silk waveguides, shown in both straight and wavy formation onto a borosilicate glass slide. The material is heated and melted down to be pushed with a specified force through a die. This die gives it a certain shape and it can be laid out in certain orientations, as shown in the figure. (**b**) The schematic and breakdown of the equipment set up to image the transverse face of the silk waveguides [36]. Reproduced with permission from John Wiley and Sons, 2009.

Parker et al. reported on the development of a printing technique used to create silk optical waveguides through direct ink writing. Ink writing is a process of extrusion-based 3D printing where an aqueous solution of 28–30 wt% silk is extruded through a 5 μm glass nozzle into a methanol reservoir, shown in Figure 8, as the silk waveguides are drawn across the plate [36]. The resulting printed silk waveguides crystallize in the methanol coagulation reservoir and retain the rod-shaped morphology. The reservoir induces a structural shift in the silk, transforming them from a randomized coil formation into stiff β-sheets, which promotes the solidification of the extruded waveguides into desired shapes [36]. The silk waveguides were printed onto borosilicate glass slides in varying straight and curved configurations, and both the straight and curved silk configurations effectively guided light, proving the feasibility of weather-fabricated configuration. The direct ink writing method for 3D printing silk fibers compares well to optical-grade silk fibers and provides a combination of simplicity and functionality in fabrication.

### 4.6. Integration of Biopolymer Nanoparticles

**Figure 9:**
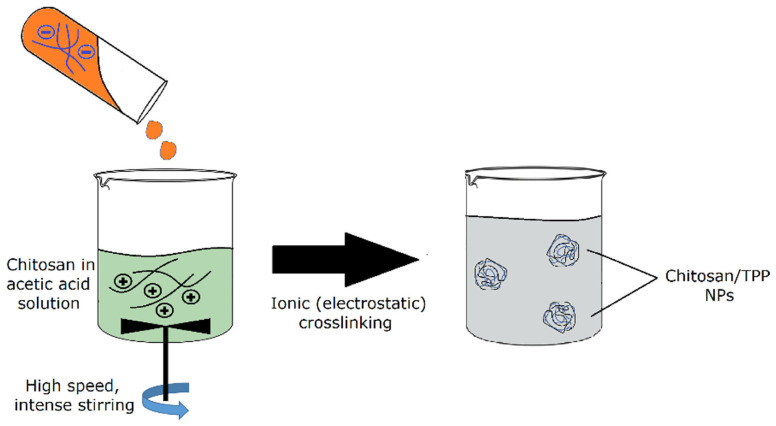
Schematic representation of ionic gelation using chitosan. The nanoparticles are added to a solvent, heated, and thoroughly mixed to produce the nanoparticle mixture. From there, it undergoes electrostatic crosslinking, and wash and centrifugation cycles follow [38] (reproduced with permission from Elsevier, 2019).

Biopolymer-based nanoparticles such as chitosan nanoparticles can be used to provide optical clarity. Coelho et al. augmented the viability of an optical implant through the incorporation of chitosan nanoparticles [37]. The method of fabrication involved the addition of chitosan to acetic acid with heat and intense stirring 12 h before filtration [37]. This method of fabrication most closely resembles spray drying, where chitosan is added to acetic acid before the nanoparticles are extruded by passing the solution through a nozzle. This is generally regarded as a simple and expedient method for synthesizing chitosan nanoparticles because it does not require separate drying steps. Another popular method of chitosan nanoparticle synthesis involving acetic acid is ionic gelation, which is shown in Figure 9. In this method of fabrication, chitosan is added to acetic acid and sodium tripolyphosphate (TPP) under intense stirring. The nanoparticles are created via ionic crosslinking. One disadvantage of this method is that it requires several post-processing steps, including washing and centrifugation cycles [38].

## 5. Applications

### 5.1. Optical Waveguides and Sensors

**Figure 10:**
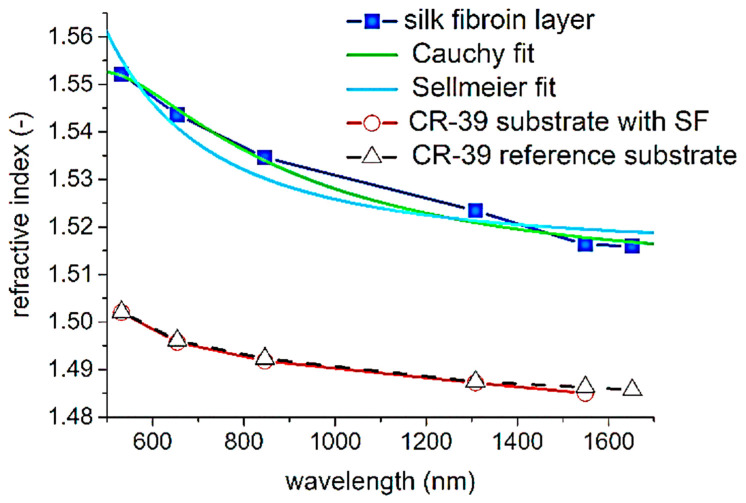
Dispersion curves of the silk fibroin layer and full construct with Cauchy and Sellmeier models [41] (reproduced with permission from Elsevier, 2021).

Generally, optical waveguides operate by propagating light and confining it within a constant cross-section area [39]. Optical waveguides are a potential solution to many problems in photomedicine and could lead to advances in implantable devices for improving diagnosis [40]. Silk fibroin already has various biomedical applications due to its biocompatibility and biodegradable properties, creating a steady rise to prominence in tissue engineering and regenerative medicine that has led to even further contemplation of this biomaterial in other fields. Some studies have considered the implementation of silk fibroin in optical waveguides for biomedical applications [41]. Prajzler et al. characterized an optical waveguide consisting of a polymer substrate and silk fibroin which was derived from *Bombyx mori* cocoons. The study consisted of preliminary efforts to chemically characterize this construct using Raman and FTIR spectroscopy. The results of these experiments indicated the successful synthesis of the construct through readings at 1513 and 1621 cm^−1^ which implied the existence of silk on the polymer substrate. The studies by Prajzler et al. also confirmed that the silk strands featured in this construct maintained 85% light transmission for wavelengths below 1110 nm, after which the transmission steadily decreased [41]. M-line spectroscopy was used to find the refractive index of the construct at various wavelengths. The results are shown in Figure 10.

Polymer waveguides are especially useful due to their transparency and flexibility [42]. Ahmed et al. discussed a new optical waveguide made from a chitosan-derived silica–phosphate nanocomposite including erbium ions for use in near-IR spectroscopy [43]. Their work to characterize this waveguide confirmed its practicality, as it was concluded that a signal of 1.36 μm led to a strong enough emission to justify the use of this platform in real-world applications [43].

Many works have demonstrated considerable success with other biopolymers, such as polylactic acid (PLA) [44,45]. Feng et al. reported the successful fabrication of a PLA copolymer-based implantable waveguide with the ability to propagate near-ultraviolet light at depths as great as 8 cm in tissue. Furthermore, Feng et al. noted that the construct had superior attenuation losses to other PLA-based waveguides reported in the literature. Finally, the viability of this intervention is compounded by its mechanical properties, which are shown to be suitable for implantation [44].

**Figure 11:**
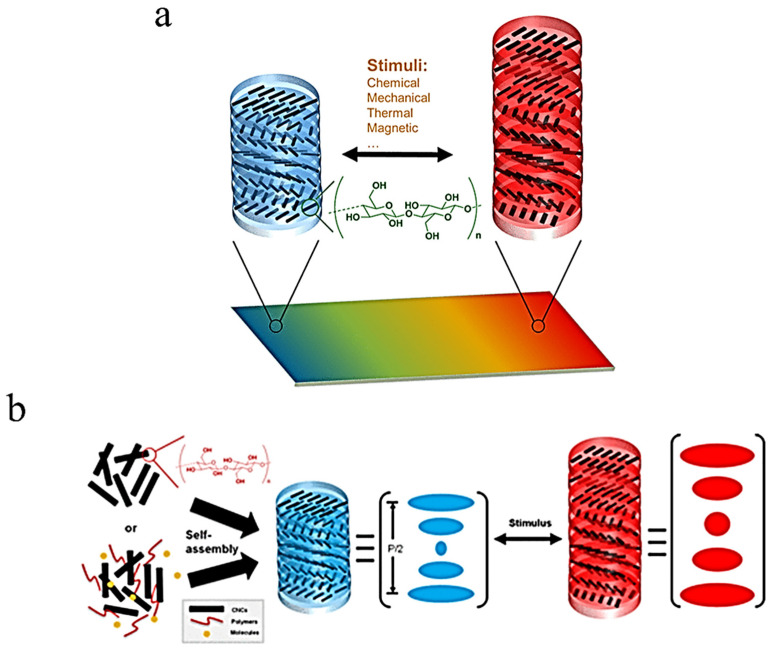
(**a**) Schematic representation of shift of CNC reflection spectrum on exposure to stimuli. (**b**) Reflection spectrum change represented by variation in helical pitch [46]. Reproduced with permission from the American Chemical Society, 2020.

Cellulose nanocrystals (CNCs) also display unique properties that enable their potential use in hygroscopic sensors for applications in medicine. Water can readily absorb into CNC pseudoplanes due to the high number of hydroxyl groups present within the CNC structures. Sufficient absorption of water molecules by the CNC has been shown to affect change within the nematic pitch and, thus, change the reflected wavelength of the CNC, as shown in Figure 11a,b [46]. The sufficient responsiveness of the sensors discussed in these applications, especially by Peng et al., and the inherent biocompatibility of cellulose nanocrystals render them as a practical option [46]. However, the applications of sensors such as these may not be limited to the human body, possibly finding use in other areas such as monitoring conditions for storage of sensitive materials. In 2016, Wu et al. also described a self-assembled photonic film made from two CNC and polyethylene glycol diacrylate (PEGDA) layers surrounding a polyamide-6 layer. At a certain wavelength, the material was reported to surpass 50% reflective intensity and achieve hyper-reflection. Due to the reflective intensity and self-assembling nature of the construct, the authors expect that this invention could find many uses beyond its hygroscopic applications [47]. The unique benefits of the biopolymers discussed in these applications, especially concerning biocompatibility, further lend evidence to their practicality.

### 5.2. Ocular Implants

Collagen has been studied extensively in various forms for an array of potential applications, many of which lie in the field of tissue engineering, due to collagen’s biocompatibility and biodegradable properties [22]. However, collagen has repeatedly demonstrated its usefulness in optics as well.

**Figure 12:**
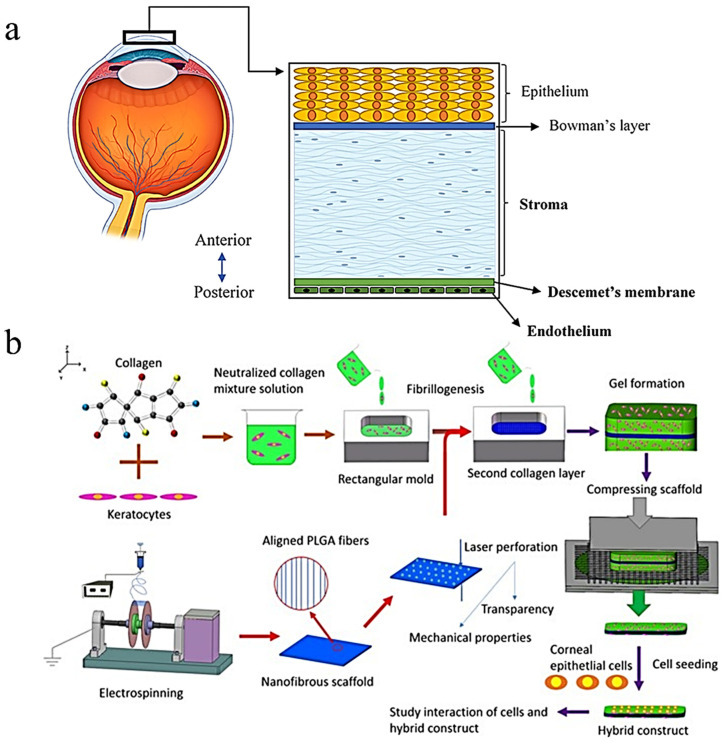
(**a**) Anatomy of the cornea [48] (reproduced with permission from Frontiers, 2021). (**b**) Method of synthesis for the ocular implant seen in Kong et al. [51] (reproduced with permission from Springer Nature, 2017).

Collagen types I and IV are the most prominent collagens found in ocular tissues and are invaluable to healthy vision due to their roles in providing structure and clarity. Song et al. described the many potential applications of these collagen types in addressing declining vision and generally poor ocular health. Among these advances is the artificial cornea, which seeks to offset the liabilities of insufficient cornea donor availability for mitigating ocular impairments such as corneal blindness [48]. The study demonstrates the feasibility of this approach in the creation of novel transplants made from seeding keratocytes in a collagen type I gel scaffold which was compressed and coated with laminin, which plays a vital role in the structural scaffolding of basement membranes [49]. In the literature, collagen hydrogels are known to have good optical clarity and light transmission due to the amount of water they contain. Beyond this, collagen hydrogels have great biocompatibility [50]. Still, this invention lacked the structural stability to be implemented in a clinical setting. Kong et al. later improved this design with the introduction of poly(lactic-co-glycolide) (PLGA) mats to supplement the strength of the compressed collagen. Perforations were induced by laser, resulting in a light transmittance of 72 ± 1.8% under 500 nm, a 15 times increase [51]. Figure 12 shows the general process that was used for the fabrication of the construct seen in Kong et al.

Recently, contact lenses have been receiving more attention as a promising avenue for ocular drug delivery. Traditional silicone-based contact lenses have been shown to induce eye discomfort after being worn for prolonged periods and novel contact lenses containing therapeutic drugs have been investigated as a potential solution; however, previous studies have shown that the incorporation of drugs within bacterial cellulose lenses can reduce the transparency of the lenses [26,37]. Coelho et al. sought to correct this defect in bacterial-cellulose-derived contact lenses by coating them with aluminum alkoxide and glycidoxypropyltrimethoxysilane (GPTS) or chitosan nanoparticles. The overall toxicities of both variations of the implants were evaluated to ascertain their safety for commercial availability. It was reported that the inclusion of both coatings resulted in visually transparent contact lenses. In contrast to the GTPS-coated lenses, the chitosan-coated lenses showed no genotoxic effects, though there was a cytotoxic effect due to the incorporation of diclofenac sodium in the lenses that will require further investigation and improvement [37]. Together, the studies referenced in this section highlight the integral responsibilities of natural biopolymers in providing clarity and low immunogenicity in ocular interventions.

### 5.3. Imaging and Diagnostics

Biomaterials derived from nanocellulose show great promise in tissue engineering due to their high biocompatibility, water absorption, water retention, mechanical properties, and optical transparency [52]. The specific benefits of optical transparency in cellulose-derived biomaterials are especially important due to the role of optical transparency in in-vivo imaging. Specifically, the optical properties of nanofibril cellulose (NFC) allow for greater imaging depth due to reduced light absorption and scattering [53]. Since it offers great potential, more research is needed to fully explore the breadth of opportunities afforded by cellulose-derived biomaterials in this capacity.

Previously established applications of other biopolymers, such as alginate hydrogels, include controlled drug delivery and tissue engineering; however, these gels are being recognized as promising solutions for challenges in diagnostics as well due to their optical properties. Mangalath et al. demonstrated a novel technique for immobilizing *Caenorhabditis elegans*, an organism that has previously been examined to study interactions between olfactory stimuli and calcium dynamics in neurons [54].

**Figure 13:**
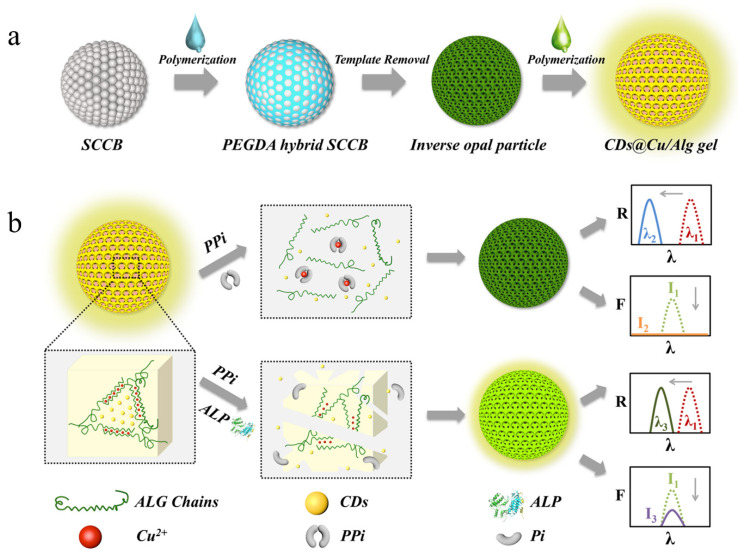
(**a**) Synthesis process for the hybrid photonic nanoparticles reported in Wang et al. [56]. (**b**) Mechanism of ALP detection by nanoparticles proposed by Wang et al. [56]. Reproduced with permission from Springer Nature, 2022.

Alginate’s optical properties allow for its use in novel diagnostic solutions. Alkaline phosphatase (ALP) is an enzyme that is widely pervasive in the human body and maintains several important roles due to its ability to hydrolyze hexose monophosphoric acid into phosphoric acid, causing it to be an integral part of bone mineralization [55]. Abnormal ALP levels have been linked to a myriad of disorders, including those of the liver and bone tissues [56]. Wang et al. described a novel platform for detecting ALP using nanoparticles composed of poly(ethylene glycol) diacrylate scaffolding with alginate hydrogels. The proposed method of detection involved the disruption of the alginate hydrogel by pyrophosphatase ions, an intermediate product of ALP activity. The disruption of the alginate hydrogel caused a phase transition and reflection spectrum shift that was successfully used to quantify the prevalence of ALP [56]. The general method of synthesis is shown in Figure 13a, which consists of the creation of inverse opal particles from template silica colloidal crystal beads and their subsequent polymerization with an aqueous alginate solution. Figure 13b depicts the general method of ALP detection using the synthesized particles.

In bioimaging and medicine, magnetic resonance imaging (MRI) is an extremely prominent technology due to its versatility in diagnostics through its ability to help diagnose a variety of conditions, including cancer. There are some concerns regarding the use of traditional metal-based contrast fluids due to the potential for their retention by tissues throughout the body and the risk of Gd(III) ions giving way to polysaccharide biopolymers rising to prominence as a potential alternative to metal-based contrast fluids in chemical exchange saturation transfer (CEST) [15,57].

Biopolymers are also enabling noninvasive bioimaging through optical tracing. The specific benefits of optical tracing via biopolymer near-infrared fluorescence (NIRF) probes include a higher in vivo half-life, superior sensitivity, and low toxicity [24]. Popular organic biopolymer NIRF probes include optically quenched biopolymers. These copolymer probes generate a strong NIRF signal when a lysine group is cleaved by an enzyme such as trypsin, causing a dramatic increase in NIRF signal, even allowing for small tumors to be reliably detected [24]. These probes are compatible with a variety of imaging technologies such as fluorescent endoscopic imaging (FEI). FEI is an application of basic fluorescence reflectance imaging in which a camera, using an optical bandpass filter, selectively captures fluorescent light [58]. These biopolymer probes are uptaken and retained by tumors due to the enhanced permeability and retention effect. This phenomenon exists due to the anatomical deformities present in tumors which enable macromolecules to be retained by these tissues far more easily than others. As such, organic biopolymer NIRF probes of this description may be useful in diagnosing various cancers.

### 5.4. Optical Fibers

Traditionally, silica-based optical fibers have been used in a variety of applications and contexts far beyond medicine, such as in telecommunication services and infrastructure. However, polymers have also been used in optical fibers and have the unique benefits of a higher diameter due to their multimode capabilities, enabling more ease of use in designing systems with this type of optical fiber [59]. Unfortunately, polymer optical fibers suffer from two crippling drawbacks. First, the attenuation properties associated with biopolymer optical fibers are less desirable than silica-based fibers. Specifically, the inherent attenuation of polymer optical fibers is far greater than silica optical fibers and rises with wavelength, relegating polymer optical fibers to shorter systems where their ability to transmit light is not greatly affected by this shortcoming [59]. Second, most polymers found in polymer optical fibers are derived from environmentally hazardous sources such as fossil fuels [59]. In these two drawbacks, there is an apparent need for innovation.

**Figure 14:**
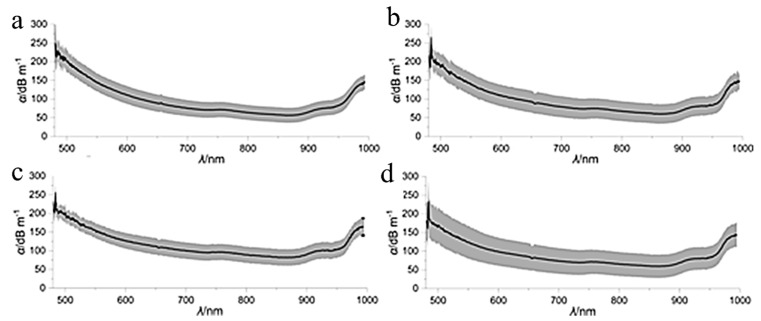
Attenuation loss spectra of cellulose optical fibers with different cladding materials: (**a**) bare core fiber; (**b**) cellulose diacetate cladding; (**c**) cellulose acetate propionate cladding; and (**d**) cellulose acetate butyrate cladding [60] (reproduced with permission from the American Chemical Society, 2021).

Reimer et al. investigated the potential of a biopolymer optical fiber containing microcrystalline cellulose (MCC). Cellulose fibers served as the cores of these optical fibers and were obtained through wet-spinning and continuous drying for 3 days. The attenuation loss of the biopolymer optical fiber was evaluated using the nondestructive substitution method with three different cellulose-based cladding materials, shown in Figure 14 which shows the attenuation loss of the three cladding materials at various wavelengths [60]. Each of these experimental cladding materials shows an increase in attenuation and standard deviation relative to the bare fiber. The attenuation losses of these fibers are higher than those composed of synthetic polymers, failing to meet the expectations that the attenuation losses would instead be lower. To explain these results, Reimer et al. clarified that the method of synthesis used for the cellulose biopolymer optical fibers could have increased the extrinsic attenuation of this optical fiber by introducing variability in the core diameter. These inadequacies led the authors to call for further research to optimize the method of synthesis seen in the study. Ultimately, while it is necessary to investigate alternative designs for optical fibers, Reimer et al. demonstrated that some obstacles may still be present in their fabrication [60].

The properties of cellulose enable countless potential applications in medicine, including novel polymer optical fibers with increased functionality. Dupuis et al. reported the successful fabrication of an optical fiber consisting of cellulose butyrate tubes separated by hydroxypropyl cellulose powder. This construct was found to have a transmission loss of 1 dB⁄cm before the inner cladding structure was filled with a solution of water and HPC, which increased fiber transmission. It was also reported that the dual-core nature of the construct potentially allows for drug-release functionality [29,61]. Some of the literature speculates on the potential use of similar interventions in an intracranial context. Fu et al. reported the fabrication of biodegradable PLLA fibers for intracranial light propagation in phototherapy while making use of the superior flexibility and optical properties of PLLA. Fu et al. noted that this construct can be expanded to accommodate drug delivery with the incorporation of hollow channels in the design of these fibers.

**Figure 15:**
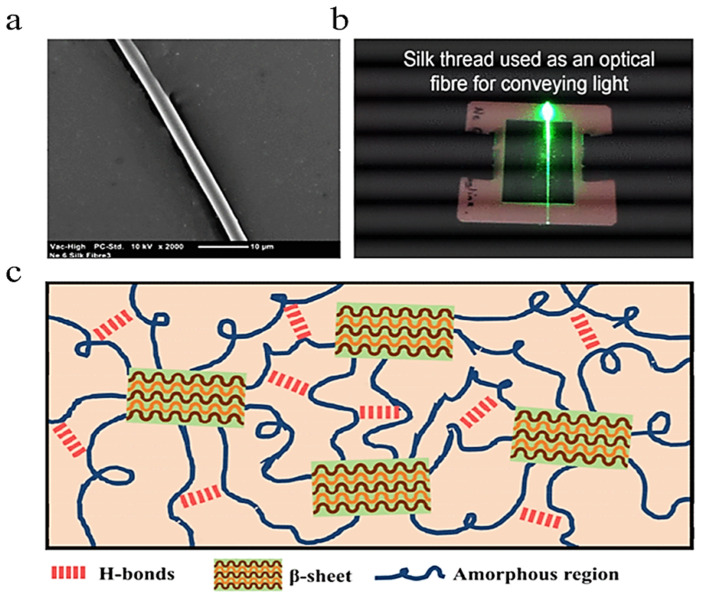
Characterization of spider silk for applications in optical fibers. (**a**) Scanning electron microscope (SEM) image of a dragline silk fiber. (**b**) Light guiding achieved in a single strand of pristine dragline silk. (**c**) Schematic representation of silk protein structures [62]. Reproduced with permission from the Institute of Electrical and Electronics Engineers, 2018.

Tow et al. reported the potential viability of spider silk as a component in chemically sensitive optical fibers. The spider silk discussed in the article is notable for its unique composition in the inclusion of crystalline β-sheet blocks among an otherwise amorphous polymer network, as shown in Figure 15c. Figure 15a shows an SEM image of the fiber and Figure 15b demonstrates the fiber serving as a medium for light propagation. The chemical properties of the crystalline regions, and the optical changes induced by chemical interactions with other molecules, cause this material to be sensitive to a variety of potential stimuli, including humidity [62]. The optical properties of *N. clavipes* spider silk were also measured, and the propagation losses of this fiber were shown to be an order of magnitude less than degummed silkworm, lending to its promise in this particular application.

## 6. Conclusions

Biopolymers are increasingly favored in optical applications, thanks to their inherent biocompatibility and biodegradability. These properties eliminate the need for surgical removal and concern over immune responses in a host, concerns that exist for some synthetic polymers. In addition, they possess an advantage in sustainability relative to synthetic polymers, whose manufacturing processes are based in oil and are environmentally harsh. These combined advantages prove their potential as synthetic polymer replacements soon. Biopolymer fabrication methods are indeed both cost-effective and low in energy demands relative to synthetic polymers, due to the lack of the need to manufacture the biopolymers. Indeed, fabrication only requires customization and modification of the existing biopolymers. The cost-effectiveness of these biopolymers, specifically, represents a major benefit over synthetic polymers and may be one of many driving forces for their overall viability and competitiveness [63,64,65]. Of all biopolymers, the most frequently researched and utilized are polysaccharide and protein biopolymers, specifically cellulose, chitin and chitosan, alginate, silk fibroin, and collagen and gelatin, due to their relative abundance and optical properties. Their customizability, both in their extraction and fabrication methods, in combination with their favorable optical properties, allows for design of specific mechanical, chemical, and optical properties in a wide variety of optical elements and applications. Current technology is directed at optimizing optical waveguides and sensors, ocular implants, imaging, and optical fibers due to failures of synthetic polymers in either sustainability, biodegradability, or biocompatibility. Therefore, biopolymers possess the potential to serve as cost-effective and safe optical materials in current and future biomedical applications. Towards future applications, the next steps in their research will include researching additional capabilities of biopolymers in optical applications and validating their potential in real-world studies, the perfection of biopolymer extraction and fabrication methods to preserve properties and include additional ones, and the preservation of properties upon degradation inside the body. Once these areas have been further explored and perfected, the biopolymers will be suitable to overtake synthetic polymers in optical applications, both serving as replacements for current applications and also creating opportunities for more. In conclusion, the rapidly expanding realm of biopolymers in optics epitomizes an uncharted territory teeming with possibilities, necessitating extensive investigative endeavors to unveil their complete range of applications and undiscovered prospects.

## Figures and Tables

**Table 1:** A summary of the fabrication of biopolymers for optical devices, including their benefits.

Method	Materials	Description	Benefits	Disadvantages
Thermal Drawing	– Utilizes cellulose [29].	– Melts material down and pulls into desired fiber shapes [29].	– Lower loss of biodegradable polymer [29]. – Minimal optical loss [29].	– Requires a preformed optical fiber [29]. – Heat can lead to polymer damage [29].
Mold Casting	– Can be utilized with any of the mentioned biopolymers.	– Pours liquid fibers into custom molds using temperature gel-based technology [2,30]. – Can be performed with glass-based, hydrogel and other temperature-based polymers [30].	– Simple and lower cost [2]. – Produces large quantity of fibers at varying lengths [30].	– Requires treatment to remove water or other possible contaminants [30]. – Needs specifically shaped mold [30].
Dry-Jet Wet Spinning	– Can be utilized with any of the discussed biopolymers.	– Pumps liquid polymer into a coagulation bath [31,32] – Wheels pull the fibers out of the bath to dry while being shaped [32]	– Increases strength and stiffness of fibers [31]. – Can be customized with different materials for varying properties [32].	– Solvent can lead to polymer damage [31]. – More complex than other fabrication methods [31].
Hydrogels	– Can be utilized with any of the discussed biopolymers, although alginate is frequently utilized [33].	– Polymer matrices composed of various synthetic fibers, such as alginate [34,35]. – Forms by physical or chemical cross-linking [34]. – Uses their varying hydrophilic groups to swell with water [34].	– Can swell and react independently based on environmental factors [34]. – Absorbs high water content. – Soft and malleable, able to mimic human tissue [34].	– Cross-linking or initiation methods could lead to polymer damage [34].
Extrusion and Printing	– Frequently utilizes various silk compounds [2,36].	– Molten polymer is forced into a die with a specified pattern to form a desired shape [2]. – Polymerization and formation of starting material occurs inside reactor [2]. – Uses high amounts of force and temperature to dispense material through a micronozzle [2].	– Batch-extrusion allows for higher processing productivity [2]. – Highly customizable based on shape of die and porosity [2].	– High operating temperatures that can lead to polymer damage [2].

## Data Availability

Not applicable.
